# Impact of Beta-blocker, ACE Inhibitor, and ARB therapy on thoracic aorta wall shear stress in bicuspid aortic valve patients

**DOI:** 10.1186/1532-429X-18-S1-P345

**Published:** 2016-01-27

**Authors:** Katherine McGee, Emilie Bollache, Alex J Barker, James C Carr, Michael Markl, Preeti Kansal

**Affiliations:** 1 Radiology, Northwestern University Feinberg School of Medicine, Chicago, IL USA; 2 Cardiology, Northwestern Memorial Hospital, Chicago, IL USA

## Background

Bicuspid aortic valves (BAV) are associated with higher incidence of aortopathy such as aortic dilatation, dissection, and aortic valve stenosis. Recent 4D flow MRI studies have provided evidence that BAV aortopathy is associated with specific hemodynamic abnormalities and increased wall shear stress (WSS) which is thought to alter aortic endothelial cell function and induce remodeling and dilatation. Pharmacologic management of BAV is currently debated. It has been hypothesized that blood pressure control using medical therapy can decrease or reduce the rate of change to central arterial pressure and thus reduce wall forces such as wall shear stress (WSS) on the aneurysmal segment of the aorta to prevent aortic remodeling and dilatation. However, in-vivo studies investigating the effect of cardiac medications on aortic WSS in BAV patients are lacking. In this longitudinal follow-up 4D flow MRI study, the effects of Beta blockers (BB), Angiotensin converting enzyme inhibitors (ACEI), and Angiotensin II Receptor Blockers (ARB) on the aortic WSS was investigated using 4D Flow MRI.

## Methods

**Figure 1:**
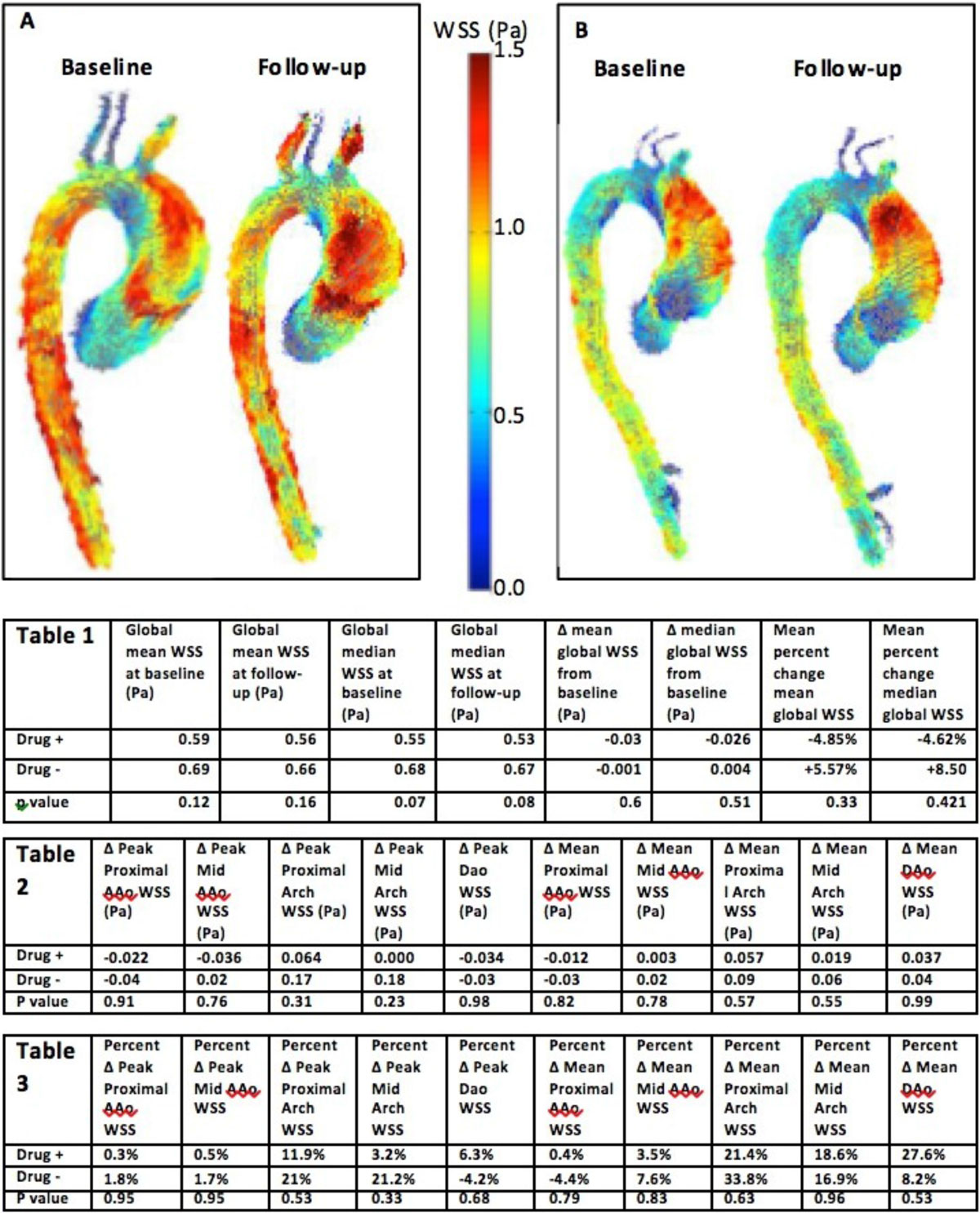
**A: global WSS map of thoracic aorta of subject not on cardiac medication**. B: global WSS map of thoracic aorta of subject prescribed BB prior to follow-up. Table 1: Global WSS findings. Table 2: Changes in raw WSS from baseline to follow-up. Table 3: Percent change from baseline. AAo: Ascending aorta; Dao: Descending Aorta.

13 patients referred for MRI to assess aortic valve function and morphology were retrospectively included. MRI included 4D flow MRI (time resolved 3D PC MRI with 3-directional velocity encoding) to assess in vivo blood flow velocities with full volumetric coverage of the thoracic aorta. Each participant underwent baseline and a 6-19 (10.7 ± 3.5 months) month follow-up cardiac MRI including 4D flow MRI. The first group ("Drug+" n = 5) underwent a baseline scan without ARB, ACEI, or BB and was prescribed one such medication prior to the follow-up scan. The second group ("Drug-" n = 8) was prescribed no such medical therapy for both study visits. Data analysis included the 3D segmentation of the thoracic aorta (MIMICS, Materlise, Belgium) and calculation of systolic 3D WSS along the entire aortic wall from 4D flow velocity acquisition as shown in Figure 1, panel A and B. Mean and median WSS was quantified in the proximal ascending aorta, mid ascending aorta, proximal arch, mid arch, and descending aorta.

## Results

Summarized in Table 1, the prescription of medical therapy did not result in significant changes in global (entire aorta, table 1) or regional (aortic segments, tables 2 and 3) WSS. Changes in mean global WSS were small and similar for the Drug+ group 0.59 ± 0.07 Pa vs. 0.56 ± 0.08 Pa, -4.9% differences, p = 0.33) and the Drug- group (0.69 ± 0.13 Pa vs. 0.66 ± 0.15 Pa, 8.5% differences, p = 0.421).

## Conclusions

Overall there was a small non-significant 4.9% decrease in mean global WSS from baseline in patients prescribed ACEI, ARB, or BB. WSS was not statistically significant between the two patient groups. Our findings suggest that current medical therapy is not effective in changing aortic hemodynamics and reducing systolic WSS. Further investigation in larger cohorts is necessary to confirm these findings and determine if WSS measurements can be used to evaluate the effectiveness of drug therapy.

